# Friction Reduction for a Rotational Gyroscope with Mechanical Support by Fabrication of a Biomimetic Superhydrophobic Surface on a Ball-Disk Shaped Rotor and the Application of a Water Film Bearing

**DOI:** 10.3390/mi8070223

**Published:** 2017-07-17

**Authors:** Dianzhong Chen, Xiaowei Liu, Haifeng Zhang, Hai Li, Rui Weng, Ling Li, Zhongzhao Zhang

**Affiliations:** 1 MEMS Center, Harbin Institute of Technology, Harbin 150001, China; dc2e12@163.com (D.C.); lxw@hit.edu.cn (X.L.); lihai7772006@126.com (H.L.); hit00@126.com (R.W.); linglimems@hit.edu.cn (L.L.); 2 State Key Laboratory of Urban Water Resource & Environment, Harbin Institute of Technology, Harbin 150001, China; 3 Key Laboratory of Micro-Systems and Micro-Structures Manufacturing, Ministry of Education, Harbin 150001, China; 4 Communication Research Center, Harbin Institute of Technology, Harbin 150001, China; zzzhang@hope.hit.edu.cn

**Keywords:** lotus leaf, superhydrophobic surface, ball-disk shaped rotor, water film bearing, rotational gyroscope

## Abstract

Friction between contacting surfaces of metal materials restricts the application of mechanical support in the high-precision inertial device of a rotational gyroscope. Instead, a disk- or ring-shaped rotor is electrostatically or magnetically suspended. However, stability of the rotor suspension restricts further improvement of the measurement precision. In the developed rotational gyroscope, a stable mechanical rotor supporting scheme with low friction is achieved by fabrication of a superhydrophobic surface with similar nanostructures of the lotus leaf on the carbon steel ball of the ball-disk-shaped rotor and the addition of a water film between the rotor ball and bronze hemispherical supporting bowl, which forms a water film bearing. The special design of the ball-disk-shaped rotor makes it possible for the application of a low-friction water bearing in the gyroscope, with rotor tilting motion. With a superhydrophobic surface, friction is further decreased and the rated spinning speed increases 12.4%, resulting in approximately the same proportion of increase in the scale factor. Moreover, superhydrophobic surface reduces mechanical damping torque for precessional motion to one order smaller than electrostatic feedback torque. Thus, through close-loop control, stable damping characteristics for precessional motion are obtained. The gyroscope exhibits excellent performance with the parameters of the measurement range, scale factor, nonlinearity, resolution, bias stability, and dynamic setting time tested to be −30°/s to 30°/s, −0.0985 V/(°/s), 0.43%, 0.1°/s, 0.5°/h, 0.1 s, respectively.

## 1. Introduction

In traditional levitated rotational gyroscopes, such as the magnetically suspended gyroscope (MSG) or electrostatically suspended gyroscope (ESG), a disk- or ring-shaped rotor is suspended by magnetic or electrostatic forces, and actuated to a high spinning rate to detect the input angular speed in the radial direction [1,2]. Shearwood et al first realized the electromagnetic levitation structure and developed an MSG, in which the Al rotor is suspended by the electromagnetic repulsion force from the stator coil with the provided high-frequency AC current [3,4]. Afterwards, MSGs based on the rotor suspension principle of electromagnetic appeal between the stator coil and the high magnetic-conductivity rotor, the principle of diamagnetic repulsion between the permanent magnet and diamagnetic rotor, respectively, was developed, [5,6,7]. An ESG with a ring-shaped rotor, and symmetrically-arranged electrodes around the rotor for actuation and capacitive detection was proposed by Murakoshi in 2003 [8]. Tsinghua University designed an improved ring-shaped rotor ESG with a three-phase variable-capacitance motor [9]. Southampton University developed an ESG employing a disk-shaped proof mass, with suspension and spin electrodes on the top and bottom of the disk [10,11,12]. Various structures of ESGs and MSGs have been designed with improved rotor suspension schemes [1,13], driving methods [14,15], pick-up circuit [16,17], and control electronics [18,19,20,21]. However, stable rotor suspension is still a challenge, which restricts the measurement precision. Traditional contacting mechanical bearings can provide stable support, obviously, while, friction consumes much of the energy for rotor driving and results in unstable dynamic characteristics in precessional motion. A water bearing, as a possible substitute to the mechanical bearing with low friction, has been reported recently. Yoxall et al. designed a rotational stage with 100–300-μm-thickness satellite droplets as a liquid bearing, which obtained the spinning speed of 2400 rpm [22]. Sun et al. reported a rotational stage, utilizing ionic liquid rings as the bearing, which constructed an electric connection between the rotor and substrate as well [23], while the droplet bearing has static resistance to spinning motion, derived from the shear restoring force when there is contact angle hysteresis with the droplet. The maximum spinning speed obtained is only 2400 rpm. A liquid ring bearing, with no static resistance, has the drawback of impossibility for application in devices with rotor (disk-shaped) tilting, such as gyroscopes, caused by the restriction of the structure. A capillary motor, placing the plate rotor on a large droplet, was proposed in [24]. However, nonuniformity of the hydrophobic layer, which confines the edge of the droplet, results in large uncertainty of the rotor position (2°). Thus, the structure of one rotor-sized droplet as a bearing is not suitable for high-precision gyroscopes.

Biological structures are the result of long-term natural evolution and possess unique characteristics, such as survival mechanisms. Mimicking such structures is a shortcut to develop man-made spare-parts or systems to realize similar functions [25]. Superhydrophobicity of lotus leaves drew the attention of scientists and studies reveal that the superhydrophobic characteristic is brought about by the inherent structures of the micro-scaled papillae and superficial epicuticular wax [26]. Superhydrophobic surfaces have been applied in applications of water collection, bio-ion channel, antifouling, energy harvesting system, and corrosion resistance of metals [27,28,29]. Fluidic drag reduction, as an important branch of application, has been explored both theoretically and experimentally in recent years [30,31,32,33,34]. The discovery inspired the idea of fabricating a superhydrophobic surface on the rotor to realize a low-friction liquid bearing with a water film. The water film bearing has no problem of static resistance to the rotor spinning and the drawback of rotor tilting in the liquid ring bearing is overcome by the structure design of the ball-disk-shaped rotor, instead of the disk-shaped rotor.

In the proposed gyroscope, a carbon steel ball is fabricated at the center of a radially-magnetized ring disk as the rotor. The rotor is suspended by the magnetic interaction force between the magnetized rotor disk and the stator of the soft magnet material. Translational motion of the rotor at the stator plane is restricted by the lower pillar with a hemispherical supporting surface, forming a ball-joint, instead of levitation control system as in ESG and MSG. To decrease the influence of friction in the mechanical ball-joint, the superhydrophobic surface is fabricated on the rotor ball and water is injected into the cavity of the lower supporting pillar to form a water film bearing, thus turning dry friction between metal surfaces to friction between the fabricated superhydrophobic surface and the water film.

The fabrication process of the superhydrophobic surface on the carbon steel ball of the rotor and the structure of the designed rotational gyroscope are introduced in Section 2. In Section 3, characterization of superhydrophobic surface is observed and studied. Performance improvements by the superhydrophobic surface are analyzed and the performance parameters are tested through experiments. Finally, Section 4 concludes the significance of fabricating the superhydrophobic surface and the application of the water film bearing for friction reduction in a rotational gyroscope.

## 2. Materials and Methods

### 2.1. Materials

Chemical reagents of acetone, hydrochloric acid, potassium chloride, ethanol, and fluorinated silane were purchased from Sinopharm Chemical Reagent Co., Ltd (Shanghai, China).

### 2.2. Fabrication of the Superhydrophobic Surface

A carbon steel ball with the radius of 1.5 mm was sequentially polished with 800#, 1200#, and 1700# sandpapers. Then, it was cleaned ultrasonically in acetone, washed in deionized water for degreasing, and dried in atmosphere conditions. A solution of 0.2 M potassium chloride (KCl) was mixed with a hydrochloric acid solution of (HCl) to adjust the PH value to around 3 with the indication of a pH paper. The mixed solution (200 mL) was poured into a glass beaker and heated to 70 °C. The carbon steel ball was immersed in it and pure oxygen introduced at a rate of 160 sccm to accelerate oxidation to form a lotus-leaf-like nanostructure of Fe_3_O_4_. A magnetic bar stirred the solution at the rate of 120 rpm to promote the dissolution of oxygen bubbles. After oxidation, the ball was washed in 50 mL distilled water, dried in a N_2_ environment, and modified in an ethanol solution of fluorinated silane of 0.5 wt % for 30 min. Finally, after drying in a vacuum oven at 120 °C for an hour, superhydrophobic surface was fabricated on the rotor ball.

### 2.3. Characterization Method

A field-emission scanning electron microscope (FE-SEM, VEGA 3, TESCAN, Brno-Kohoutovice, Czech) was utilized to observe the morphology of superhydrophobic surface. Element constitution and element valence were measured by energy-dispersive X-ray spectroscopy (EDS, EX-250, Horiba Ltd, Kyoto, Japan) and X-ray diffraction (XRD, D/MAX-2000, Rigaku, Tokyo, Japan) patterns. Contact angles (CAs) of 5 μL water droplets on the superhydrophobic carbon steel sheet under the same treatment process were tested by an optical contact-angle meter system (JC2000D, POWEREACH, Shanghai, China). The drag reduction effect was valued by the static driving angle (the angle of stator magnetic potential behind rotor magnetic potential) to rotors with and without superhydrophobic surfaces under the same driving condition, the sine of which is proportional to the friction torque.

### 2.4. Gyroscope Structure

**Figure 1:**
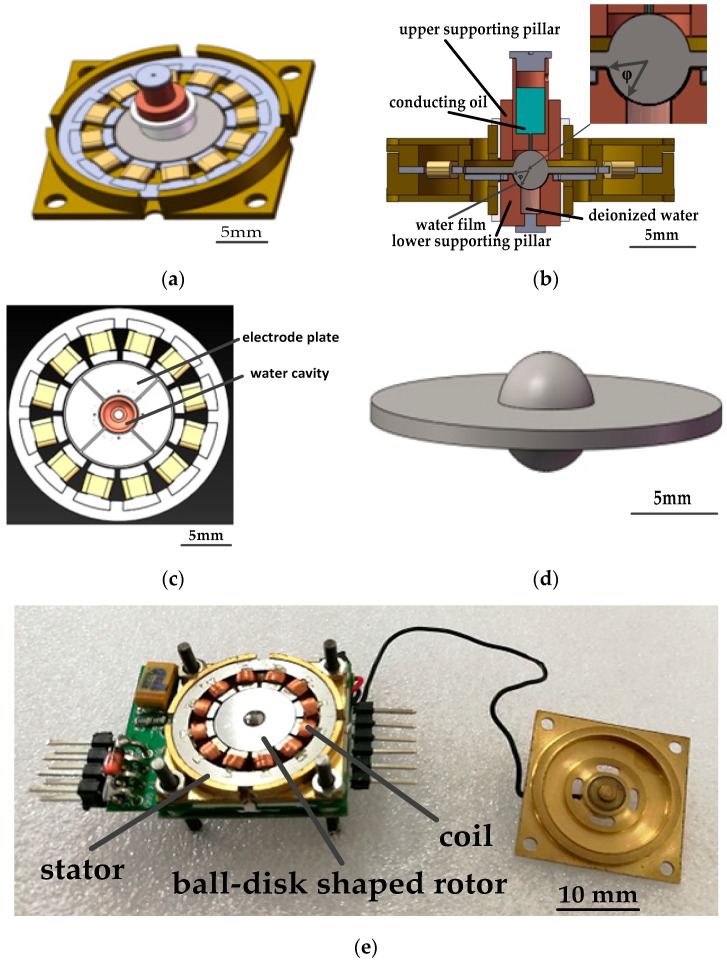
(**a**) Gyroscope structure assembly diagram without the upper frame; (**b**) Assembly cross-section diagram; (**c**) Engineering diagram of the electrode plate and water cavity; (**d**) Engineering diagram of the ball-disk-shaped rotor; (**e**) Photo of the fabricated gyroscope.

The gyroscope structure assembly diagram without the upper frame, the assembly cross-section diagram, the engineering diagram of the electrode plate and the water cavity, the engineering diagram of the ball-disk-shaped rotor, and a photo of the fabricated gyroscope are shown in Figure 1. The stator is made of silicon steel sheets with 300 coils of enameled wire around each of 12 poles. The ball-disk shaped rotor consists of a carbon steel ball, and a 2J85-grade permanent magnetic disk. The disk is magnetized unidirectionally in the radial direction. Magnetic interaction between the rotor and stator produces a rotor levitation effect. A hemispherical bowl at the lower supporting pillar limits the translational motion of the rotor in the stator plane and guarantees three rotational degrees of freedom (DOFs). A cylindrical cavity storing conducting oil is evacuated at the upper supporting pillar and a cylindrical pore is fabricated beneath, as shown in Figure 1b. Contact with weak pressure (producing little friction force) between the upper supporting pillar and the rotor ball is to establish a stable electric connection between conductive oil and the rotor ball. Deionized water is filled into a cavity in the lower supporting pillar and when the rotor is driven to a high spinning speed, deionized water forms a water film between the superhurophobic ball surface and hemispherical bowl surface. The bottom supporting pillar fixes the electrode plate (with four sectorial electrodes) under the rotor, which forms two perpendicular capacitor pairs with the rotor disk for differential detection.

## 3. Result and Discussion

### 3.1. Characterization

**Figure 2:**
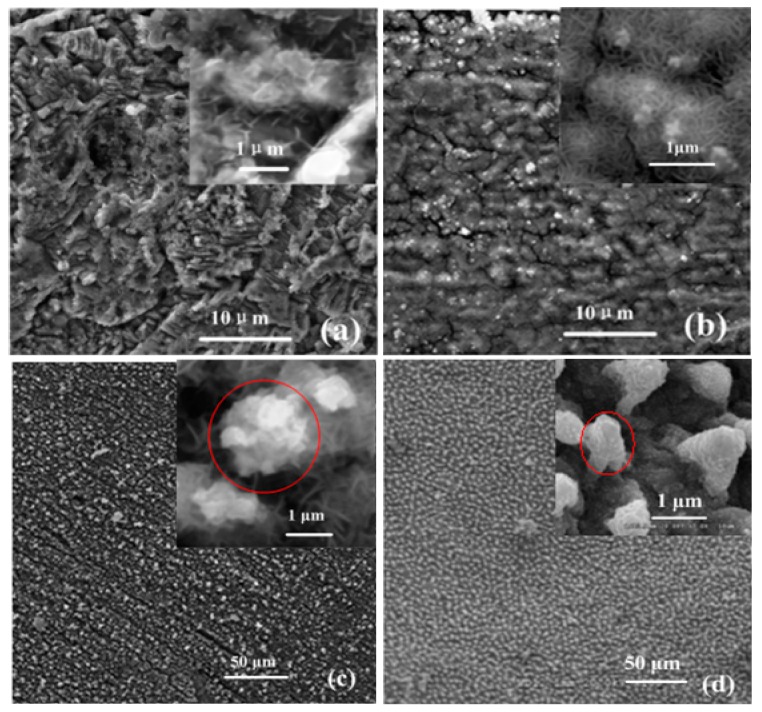
SEM images of nano-structures prepared at 70 °C in acidic solution for (**a**) 5 min; (**b**) 20 min, and (**c**) 30 min; (**d**) SEM image of the lotus leaf surface [35].

The growth process of the lotus-leaf-like structure is illustrated by SEM images of the ball surface, as shown in Figure 2. After oxidation for 5 min, nanosheets were sparsely produced. When the reaction time increased to 20 minutes, structures of nano-flowers were formed. After oxidation for another 10 min, evenly-distributed microprotrusions with diameters 2–5 μm were observed. Many nanosheets are located on each microprotrusion. The produced nano-structure on the carbon steel surface (Figure 2c) is greatly similar with the surface structure of the lotus leaf surface (Figure 2d).

**Figure 3:**
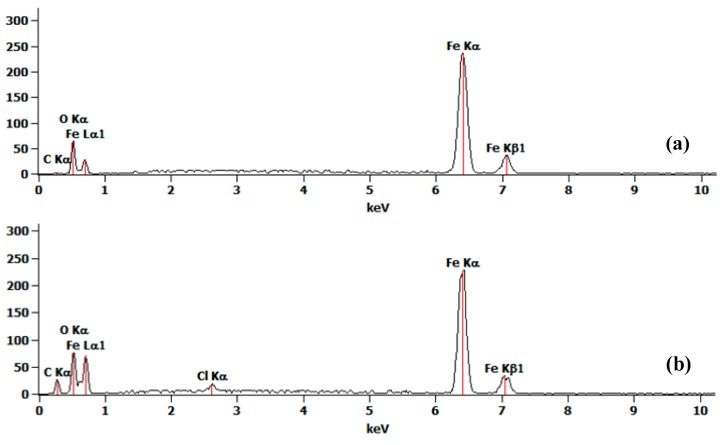

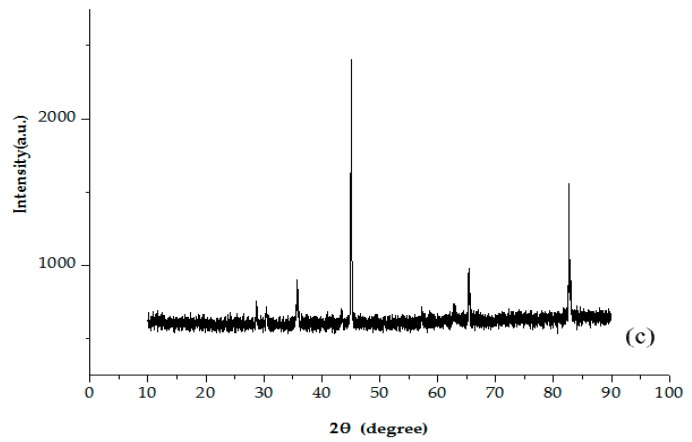
EDS spectrum of the carbon steel ball surface before (**a**) and after (**b**) oxidation; (**c**) XRD pattern of the oxidation nanosheet. Red boxes represent diffraction peaks of Fe_3_O_4_.

Energy-dispersive X-ray spectroscopy (EDS) was utilized to analyze elements of the produced superhydrophobic surface. EDS spectra of the ball surface, before and after oxidation (Figure 3a,b), indicate Fe, C, and O are the main elements, with more O after oxidation. Stronger peaks of Fe and O in Figure 3b reveal that the atomic ratio is 1:1.25. The element category and ratio satisfy the inference that the ingredients of the oxidation products is Fe_3_O_4_, pure Fe, with small amount of Fe_2_O_3_ and FeO. To further study the crystal structure of the Fe oxides, X-ray diffraction (XRD) measurement is conducted and the XRD pattern of the oxidation nanosheet is as shown in Figure 3c, which matches with the standard XRD pattern of Fe_3_O_4_. The result indicates that the main ingredient of oxidation is Fe_3_O_4_.

**Figure 4:**
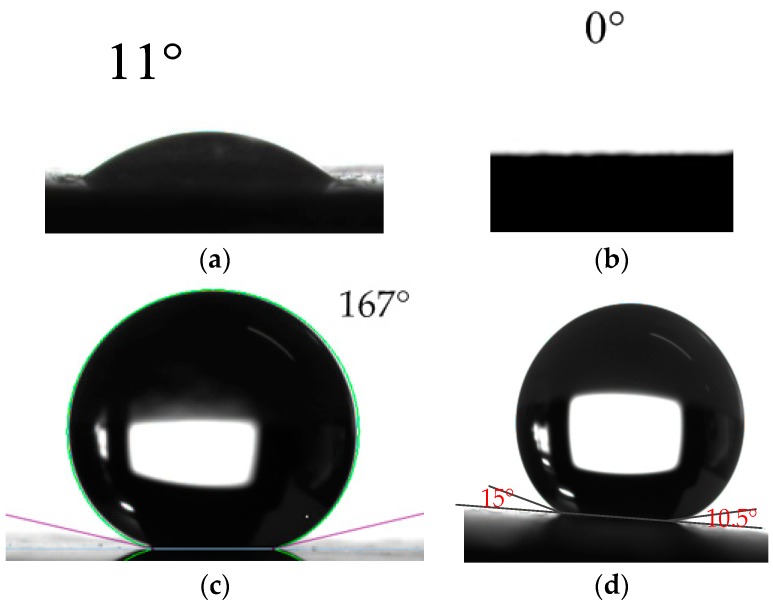
Optical photo of water droplets on surfaces with different morphology: (**a**) untreated sheet surface; (**b**) sheet surface after oxidation; and (**c**) superhydrophobic sheet surface; (**d**) Snapshot photograph of the advancing CA and receding CA of the superhydrophobic sheet surface.

Wettability of the material surface is often assessed by CA and contact angle hysteresis, which are inconvenient for the ball surface. To measure the CA and contact angle hysteresis, the surface is fabricated on a carbon steel sheet with the same preparation flow. Optical photos of water droplets on the sheet’s surface after different processing procedures with the measured CA, measured advancing CA, and receding CA of the final fabricated superhydrophobic sheet surface are shown in Figure 4. For the hydrophobic material of pure Fe, the water CA is about 11° (Figure 4a). After oxidation, the CA is reduced to 0° (Figure 4b) and the surface is rough and superhydrophilic. Finally, modification turns the surface from superhydrophilic to superhydrophobic with the CA, advancing CA, and receding CA of 167°, 15°, and 10.5° (Figure 4c,d), respectively. Then the contact angle hysteresis is 4.5° (15° − 10.5° = 4.5°). A superhydrophobic surface with the same CA and contact angle hysteresis is fabricated on the rotor ball.

### 3.2. Increase of the Rated Spinning Speed by the Superhydrophobic Surface

**Figure 5:**
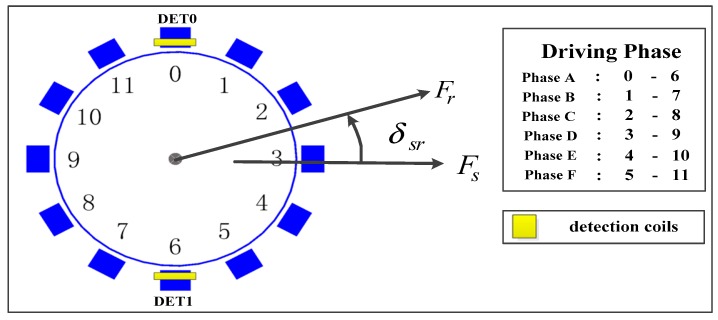
Driving schematic diagram.

**Figure 6:**
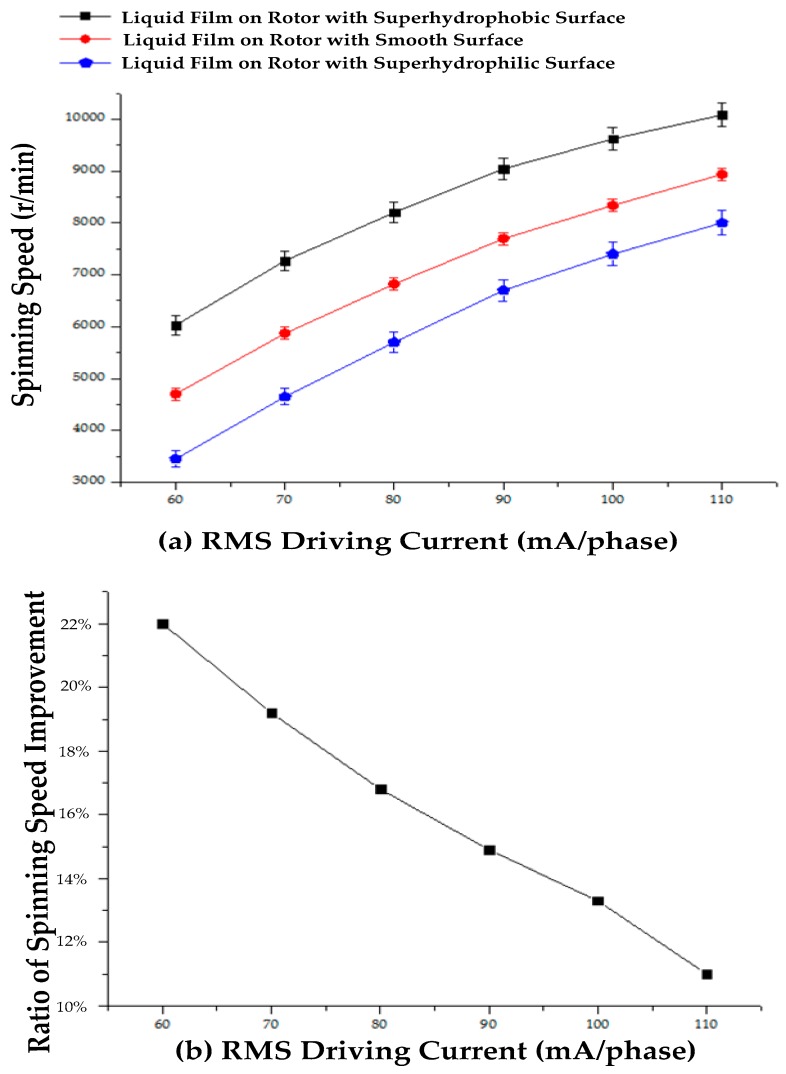
(**a**) Steady-state spinning speed under different RMS driving currents; (**b**) The ratio of the spinning speed improvement compared between rotor with smooth and superhydrophobic surfaces under different RMS driving currents.

The schematic diagram of the brushless direct current motor (BLDCM) driving scheme is shown in Figure 5. Opposite ones of 12 poles with coils around form driving pairs, from Phase A to Phase F. Back electromotive force (EMF) detection coils, marked as DET0 and DET1, are installed around the same poles with the driving pairs of Phase A. Driving a square voltage added, in turn, from Phase A to Phase F produces a magnetic field at the rotational speed of *ω_s_*. Magnetic potentials of this rotating magnetic field and the magnetized rotor are noted as *F_s_*, *F_r_*. The driving angle *δ_sr_*, which denotes the angle rotational vector *F_s_* behind *F_r_*, is detected by DET0 and DET1 and applied as the control variable at closed-loop acceleration and driving voltage adjustment stages. According to [36], the magnetic torque *T* exerted on the rotor is expressed as:

(1) $$T=-\mu_{0}F_{s}F_{r}\sin\delta_{sr}$$

where *μ*_0_ is permeability, and *F_s_* is controlled by the driving current *I_s_*. At the acceleration stage and steady-state stage, *δ_sr_* is controlled at 60° and 85°, respectively, with margins of 30° and 5° to avoid driving loss. A larger margin at the acceleration stage is due to the inaccurate driving angle estimate at a low spinning speed. At the steady-state stage, the magnetic torque *T* equals the damping torque *T_f_*, derived from the viscous force between the superhydrophobic surface and the water film bearing. Tangential viscous stress is expressed as:

(2) $$\tau =\eta \frac{v_{d}}{h}$$

where *η* is the viscosity of the water film, *v_d_* is the speed difference between water film and rotor surface, and h is the thickness of the water film. Therefore, *T_f_* is calculated as:

(3) $$T_{f}=\int_{S}\tau (\theta )R(\theta )ds=\frac{2\pi R^{3}\eta }{h}\int_{0}^{\phi }{\left(\cos\theta \right)}^{2}v_{d}(\theta )d\theta =\frac{2\pi R^{3}\eta }{h}\int_{0}^{\phi }{\left(\cos\theta \right)}^{2}{\bar{v}}_{d}d\theta$$

where *η* is the viscocity of the water film, *v_d_*(*θ*) is the speed difference between the water film and the rotor surface at a certain position, *h* is the average thickness of the water film, *R* is the rotor radius, and *ϕ* is indicated in Figure 1b. Equations (1) and (3) indicate that, at a steady-state (*T = T_f_*), when driving current *I_s_* increases, the rotor spinning speed *ω* is increased with a larger ${\bar{v}}_{d}$. With the superhydrophobic surface, the contacting surfaces between the water film and the rotor ball are turned to a Cassie state, with air bubbles captured by fabricated nano-structures. Then, some of the contacting surfaces between the carbon steel and water film is changed between air bubbles and the water film, and the integral area in Equation (3) is decreased. Thus, under the same *I_s_* and controlled *δ_sr_*, steady-state ${\bar{v}}_{d}$ is improved to maintain *T_f_*. The rotor spinning speed *ω* is increased. Steady-state spinning speeds under different RMS driving currents for rotors with the smooth surface, superhydrophilic surface, and superhydrophobic surface, supported by the water film bearing are tested, as shown in Figure 6a, with the ratio of the spinning speed improvement compared between the rotor with the smooth and superhydrophobic surfaces shown in Figure 6b. Without water, the dry rotor is not started when the driving current increases to 110 mA, for there is a large dry friction torque between the rotor ball and the hemispherical supporting bowl. When the RMS driving current per phase increases from 60 mA to the maximum allowable current of 110 mA, the rotor spinning speed increases from 6028 rpm to 10,084 rpm, 4702 rpm to 8970 rpm, and 3451 rpm to 8003 rpm for rotors with a superhydrophobic surface, smooth surface, and superhydrophilic surface (Figure 6a). Thus, friction between the rotor surface and the water film bearing is closely related to the morphology of the rotor surface. The fabricated superhydrophobic surface reduces friction between the rotor surface and the water film during the spinning motion, and obtains a higher spinning speed. The ratio of the spinning speed improvement compared between the rotor with smooth and superhydrophobic surfaces decreases from 22% to 11% with the increase in the driving current (Figure 6b), for a high rotor spinning speed will cause a larger shear force between the water film and the captured air bubbles, which washes away some of captured air bubbles, thus weakening the friction reduction effect.

### 3.3. Gyroscope Operational Principle and Performance Improvement

**Figure 7:**
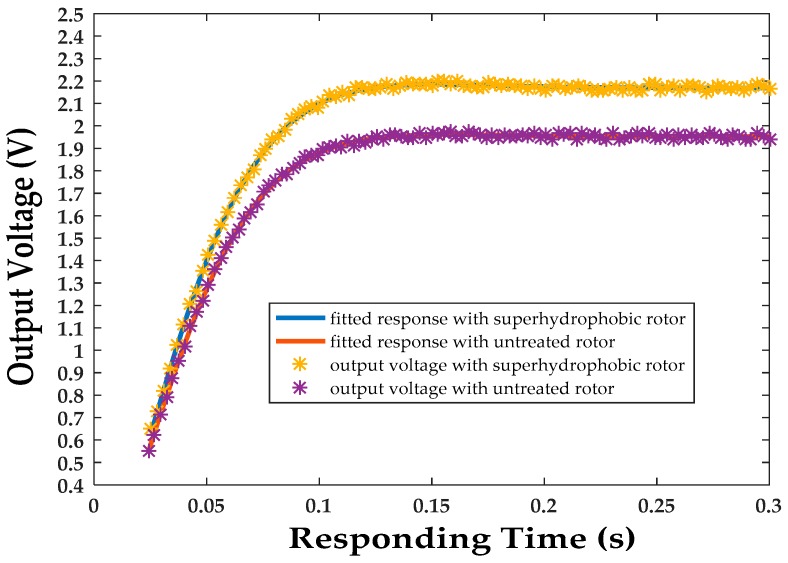
Fitted curve of the output voltage under the step angular speed input of 20°/s for gyroscopes with and without a superhydrophobic rotor.

The stator adopts the BLDCM driving mode and produces a stable rotating magnetic field in the rotor area. The ball-disk-shaped rotor is driven to a high rotational speed, with a large angular momentum *H*. The expression of *H* is:

(4) $$H=I_{z}\omega_{z}$$

where *I_z_* is the moment of inertia of the rotor and *ω_z_* is the angular speed of the rotor. *I_z_* is calculated as:

(5) $$I_{z}=∭r^{2}\rho dV=\frac{8}{15}\pi \rho_{1}R_{1}{}^{5}+\frac{1}{2}\pi \rho_{2}h(R_{2}^{4}-R_{1}^{4})$$

where *ρ*_1_, *ρ*_2_ are the density of the ball (carbon steel) and the disk (2J85-grade permanent magnet), *R*_1_, *R*_2_ are the disk inner radius (which equals the ball radius) and outer radius, and h is the disk thickness. Input angular speed *ω* at the stator plane will result in a Coriolis torque *M_G_*, expressed as:

(6) $$M_{G}=\bar{\omega }\times H$$

deflecting the rotor. Interaction between the magnetized rotor and the stator of soft magnetic material generates an elastic rotor restoring torque, proportional to deflection angle, with coefficient *C*. Two capacitor pairs detect the perpendicular deflection angles. Then, through a digital signal processing (DSP)-based proportional-derivative (PD) controller, angle signals are converted to control signals for the DC generator to produce feedback voltages, added at four electrodes, to control the damping coefficient D. The motion equations of the designed two-DOF gyroscope are:

(7) $$I\ddot{\beta }-H\omega_{y}-H\dot{\alpha }+C\beta +D\dot{\beta }=0$$

(8) $$I\ddot{\alpha }-H\omega_{x}+H\dot{\beta }+D\dot{\alpha }+C\alpha =0$$

where *I* represents moment of inertia of the rotor, *ω_x_*, *ω_y_* are perpendicular input angular speeds at the rotor plane. The fabrication of the superhydrophobic surface on the rotor ball increases the rated spinning speed, thus increasing *H*. To investigate the performance improvement, gyroscopes with smooth and superhydrophobic rotor balls, under an RMS driving current of 110 mA/phase, are put on the rate table with a step angular speed input of 20°/s. Fitted curves (Figure 7) of the output voltage reveal that larger angular momentum *H* will bring a proportional increase of steady-state output voltage (10,084 rpm/8970 rpm ≈ 2.17 V/1.95 V), while having little influence on dynamic characteristics of settling time (about 0.1 s). Therefore, fabrication of the superhydrophobic surface reduces the driving resistance, improves the rated spinning speed of the rotor, and increases the steady-state deflection angle of the rotor, which results in a larger output scale factor.

### 3.4. Gyroscope Performance Parameters

**Figure 8:**
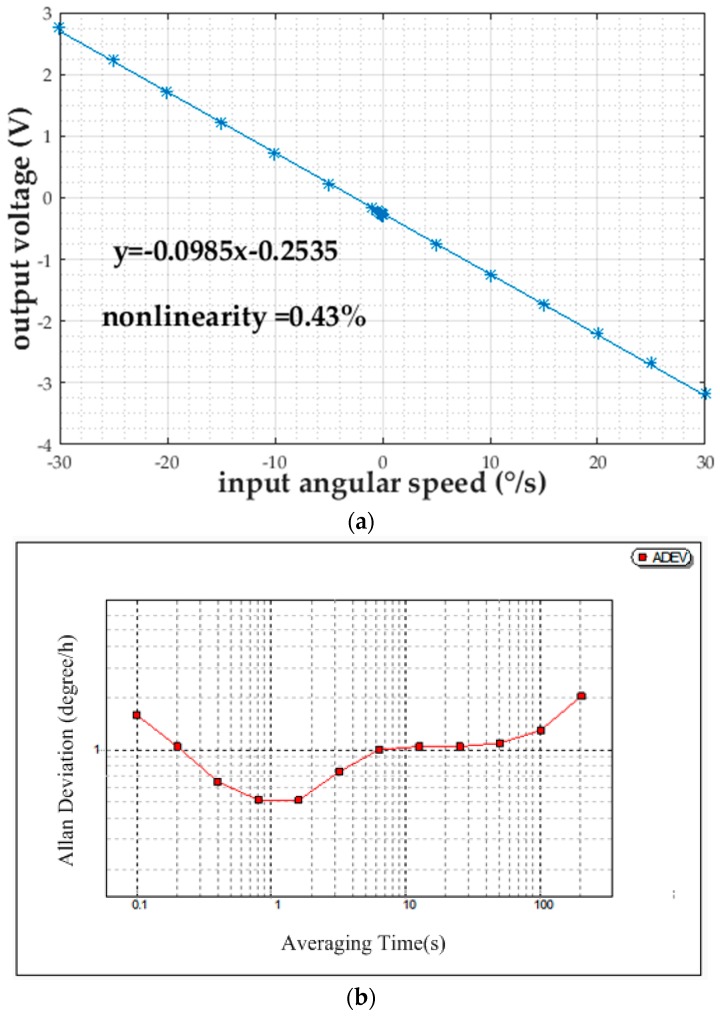
(**a**) Input-output characteristic of the proposed rotational gyroscope; (**b**) Log-log plot of ADEV versus the averaging time for the angular speed of the proposed rotational gyroscope.

The gyroscope was tested on the rate table with a rated spinning rate of 10,000 rpm. Under the input angular speeds from −30°/s to 30°/s with an interval of 5°/s and an additional nine exponentially-distributed input angular speeds from −0.00625°/s to −1.6°/s, the output voltage is recorded and linearly fitted with Matlab (R2016a, MathWorks, Natick, MA, USA), as shown in Figure 8a. The scale factor, nonlinearity, and resolution are confirmed to be −0.0985 V/(°/s), 0.43%, and 0.1°/s, respectively. The measurement range is restricted by the parallel distance between the rotor disk and the detection electrode plate, an increase of which will result in larger nonlinearity. For the bias stability test, the proposed rotational gyroscope is actuated to 10,000 rpm on the rate table with zero input angular speed for 0.5 h measurement data collection. The Allan deviation (ADEV) is plotted versus the averaging time, as shown in Figure 8b. Bias stability, the lowest value of ADEV with zero log-log slope, is 0.5°/h. The measurement performance of the proposed gyroscope is summarized in Table 1.

The proposed gyroscope exhibits excellent static measurement performance with bias stability as low as 0.5°/h and dynamic response performance with settling time of only 0.1 s. For the restriction of the applicable feedback voltage for fear of air breakdown, the distance between the rotor disk plane and the detection electrode is adjusted to 50 μm. The small distance restricts the measurement range to −30°/s~30°/s. However, for differential capacitance detection, the small electrode distance contributes to low nonlinearity of 0.43% and a high resolution of 0.1°/s.

## 4. Conclusions

A rotational gyroscope with the structure of a ball-disk-shaped rotor is developed. The structure replaces the complicated and unstable rotor suspension system in traditional rotational gyroscopes of ESG and MSG with a low-friction water film bearing. The design of the ball-disk-shaped rotor and semispherical lower supporting bowl maintains the gap between them during rotor spinning and precessional motion, making the application of the water film bearing possible. A lotus-leaf-like superhydrophobic surface, with a CA of 167° and contact angle hysteresis of 4.5°, is fabricated on the rotor surface, turning the contacting surfaces between water film and rotor ball to a Cassie state, with lower friction. Under the same driving current, the rated spinning speed increases 12.4% (8970 rpm to 10,084 rpm) compared between the rotor with and without the superhydrophobic surface, which contributes to a scale factor improvement of 11.3% with little influence on the dynamic characteristics. High performance of the proposed gyroscope with bias stability of 0.5°/h and settling time of 0.1 s suggests that fabrication of the superhydrophobic surface and the application of the water film bearing is a feasible method to realize high-precision rotational gyroscopes with low-friction mechanical support. Further work will focus on magnetic interaction between the rotor and stator to obtain a larger restoring coefficient *C* (by changing the dimension parameters of the rotor or stator, changing the magnetic material of the rotor disk, etc.), therefore, improving the measurement range.

## Figures and Tables

**Table 1:** Performance of the fabricated gyroscope.

Performance	Value (Unit)
Measurement Range	−30°/s~30°/s
Scale Factor	0.0985 V/(°/s)
Nonlinearity	0.43%
Resolution	0.1°/s
Bias Stability	0.5°/h
Settling Time	0.1 s
